# Highly sampled measurements in a controlled atmosphere at the Biosphere 2 Landscape Evolution Observatory

**DOI:** 10.1038/s41597-020-00645-5

**Published:** 2020-09-15

**Authors:** Jorge Arevalo, Xubin Zeng, Matej Durcik, Michael Sibayan, Luke Pangle, Nate Abramson, Aaron Bugaj, Wei-Ren Ng, Minseok Kim, Greg Barron-Gafford, Joost van Haren, Guo-Yue Niu, John Adams, Joaquin Ruiz, Peter A. Troch

**Affiliations:** 1 Department of Hydrology and Atmospheric Sciences, University of Arizona, 1133 James E. Rogers Way, Tucson, AZ 85721 USA; 2 Departamento de Meteorología, Universidad de Valparaíso, Av. Gran Bretaña 644, Playa Ancha, Valparaíso Chile; 3 Biosphere 2, University of Arizona, 32540 S Biosphere Road, Oracle, AZ 85623 USA; 4 Department of Astronomy/Steward Observatory, University of Arizona, 933 N Cherry Avenue, Tucson, AZ 85721 USA; 5 Department of Geosciences, Georgia State University, 38 Peachtree Center Avenue, Atlanta, GA 30303 USA; 6 Department of Geosciences, University of Arizona, 1040 E Fourth Street, Tucson, AZ 85721 USA; 7 School of Geography and Development, University of Arizona, 1064 E Lowell Street, Tucson, AZ 85721 USA; 8 Department of Soil, Water and Environmental Science, University of Arizona, 1177 E. 4th Street, Tucson, AZ 85721 USA; 9 Honors College, 1101 East Mabel Street, Tucson, AZ 18719 USA

**Keywords:** Atmospheric science, Hydrology

## Abstract

Land-atmosphere interactions at different temporal and spatial scales are important for our understanding of the Earth system and its modeling. The Landscape Evolution Observatory (LEO) at Biosphere 2, managed by the University of Arizona, hosts three nearly identical artificial bare-soil hillslopes with dimensions of 11 × 30 m^2^ (1 m depth) in a controlled and highly monitored environment within three large greenhouses. These facilities provide a unique opportunity to explore these interactions. The dataset presented here is a subset of the measurements in each LEO’s hillslopes, from 1 July 2015 to 30 June 2019 every 15 minutes, consisting of temperature, water content and heat flux of the soil (at 5 cm depth) for 12 co-located points; temperature, relative humidity and wind speed above ground at 5 locations and 5 different heights ranging from 0.25 m to 9–10 m; 3D wind at 1 location; the four components of radiation at 2 locations; spatially aggregated precipitation rates, total subsurface discharge, and relative water storage; and the measurements from a weather station outside the greenhouses.

## Abstract (summary)

**Table:** 

Measurement(s)	temperature of soil • wetness of soil • heat conductivity • atmospheric wind speed • Humidity • temperature of air • longwave radiation • shortwave radiation • atmospheric wind direction • hydrological precipitation process
Technology Type(s)	thermocouple • dielectrometry • thermopile • anemometer • weather station • pyrgeometer • pyranometer • Flowmeter Device
Factor Type(s)	replicate hillslope • location • height
Sample Characteristic - Environment	soil • atmosphere

Machine-accessible metadata file describing the reported data: 10.6084/m9.figshare.12881810

## Background & Summary

The understanding of land-atmosphere interactions is important for improvements in Earth System Modelling^1–3^ for climate assessment, weather prediction, and subseasonal-to-seasonal forecasts^4^. Although the impact of some of these interactions occur at large spatiotemporal scales affecting regional climates^5,6^ through, e.g. soil moisture - precipitation feedbacks^7,8^ and mesoscale circulations, they are primarily driven by local interactions between the land-surface and the atmospheric boundary layer^9,10^. Studies of these interactions face three major challenges^11–13^: (1) lack of observations with the adequate spatiotemporal resolution and precision^14^, (2) uncertainties due to the large number of processes and feedbacks involved, and (3) the difficulty of controlled and replicated experimentation.

In this context, the Biosphere 2 of the University of Arizona is committed to contributing to the understanding of the environment through the experimentation in several large-scale, highly-controlled and densely-monitored model ecosystems. Knowledge acquired in those experiments helps scientists to build or improve computer models representing the physical, biological, and chemical processes to be tested later in nature. These models, in turn, help guide new experiments in the Biosphere 2.

One of those model ecosystems is the Landscape Evolution Observatory^15^ (LEO). Its main goal is to better understand, through controlled experimentation, the physical, chemical, and biological processes occurring in the critical zone at the hillslope scale and their interactions with the atmosphere in the context of landscape evolution and climate change. LEO’s design^12,15–17^ was driven by the need of controlled experimentation at a larger scale than available in the past, and it was the result of a large scientific community input^18^, with a focus on interdisciplinary research.

Research in LEO has been mostly focused on the hydrological and biogeochemical processes at the hillslope scale, including water and tracer transport^19–21^, microbial patterns and soil evolution^22,23^, and bare soil carbon cycling^24,25^ but the dataset has not been extensively used for land-atmosphere interaction studies. Hence, part of the measurements in LEO are being made public to the scientific community for advancing our understanding in the microscale land-atmosphere interactions.

Atmospheric variables included in this dataset are temperature, relative humidity, wind speed, 3D components of the wind vector and the four components of radiation. Volumetric water content, heat flux, and temperature from the topmost soil layer (at 5 cm depth) are also included. Precipitation, discharge, and water storage content were measured for each hillslope and made available to complete the water related variables. Additionally, measurements from an automatic weather station outside of the bays are included as reference for the outside weather conditions. All the data are available every 15 minutes from 1 July 2015 to 30 June 2019. An automated data quality control was performed to account for missing values, expected range of measurements, outliers, and spatial and temporal consistency.

This dataset is expected to contribute to our understanding of the land-atmosphere interactions by providing a highly detailed set of measurements in a controlled environment. Science questions that could be addressed with this dataset include, but are not limited to, what is the microscale spatial variability of atmospheric and land surface states in a controlled environment? how does this microscale variability change diurnally, from day to day, and seasonally? What is the temporal relationship between the atmospheric and land surface microscale variabilities? How do atmospheric variables vary with height? What are the surface turbulent fluxes over bare soil^26,27^ through the closure of water^21^ and energy balances? what is the relationship of these turbulent fluxes with atmospheric and land surface states (e.g., the vertical gradient of atmospheric variables, the horizontal variance of near-surface atmospheric and soil variables)? It is further expected that the analysis of the existing data set can lead to new hypotheses about the interactions between the land surface and the atmosphere, and that these hypotheses can be tested through experimentation involving manipulation of environmental variables, such as rainfall and wind speed.

## Methods

### Site and instruments description

**Fig. 1:**
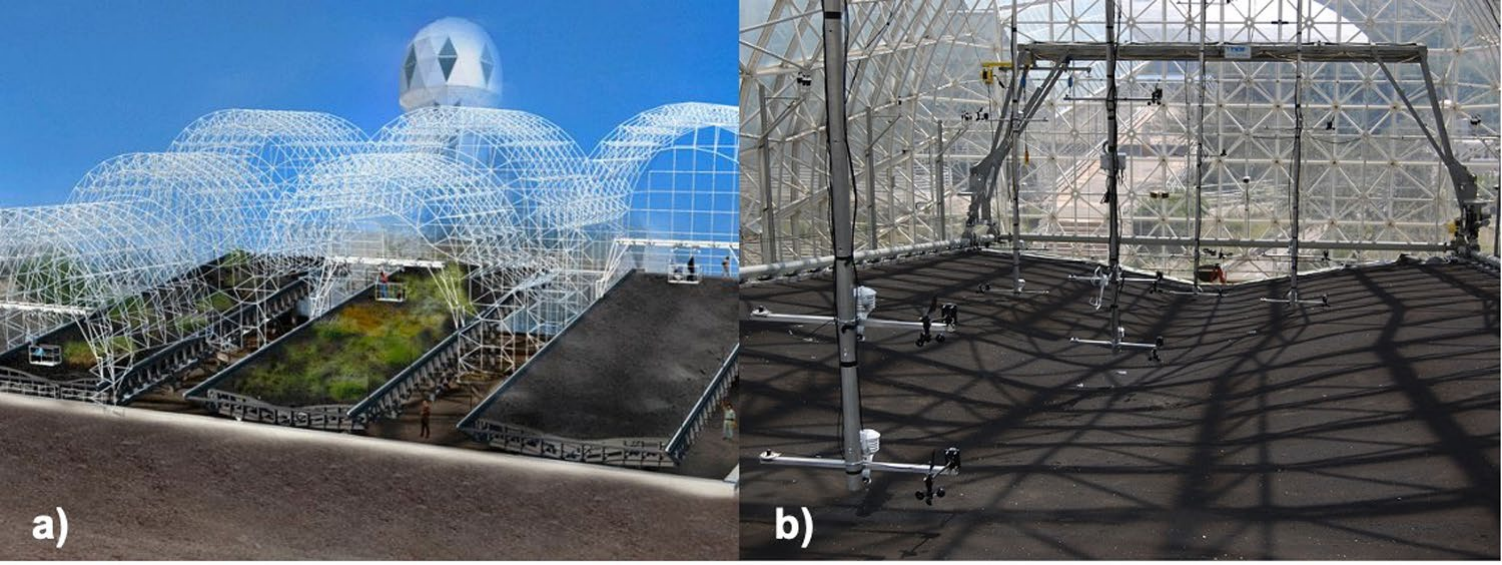
The Landscape Evolution Observatory. Schematic figure of LEO (**a**) and a picture of the above ground instrumentation in the West bay (**b**).

LEO (https://biosphere2.org/research/projects/landscape-evolution-observatory) is located at Biosphere 2, Oracle, Arizona, USA (https://biosphere2.org) and operated by the University of Arizona. It is composed of three near-identical greenhouses (Fig. 1a) covered but not sealed, by an 11 mm thick glass with an interior mylar sheet. The glass has a solar heat gain coefficient of 0.7, transmitting between 50% and 60% of total solar radiation but less than 1% of UV solar radiation^28,29^. The three greenhouses (Fig. 1a) are named East, Center and West bays, each of them containing an air volume of approximately 12,550 m^3^, 12,950 m^3^ and 12,550 m^3^, respectively; they are all facing to the south-southwest. Although the enclosed atmosphere could be highly controlled, it has been most of the time naturally driven except for precipitation during the experiments and temperature with the purpose of keeping the bays at temperatures allowing the work of the scientists.

Inside each bay there is one artificial bare soil hillslope of 11 × 30 m^2^ of surface with an average slope of 10° and 1 m average soil depth. The soil corresponds to ground basaltic tephra with a loamy sand texture and a dry bulk density of 1.5 gcm^−3^; more detailed information on soil physical and chemical properties can be found in the main article describing LEO^15^.

Buried in the soil of each hillslope are more than 1,200 sensors measuring soil water content, soil water potential, soil temperature, soil carbon dioxide concentration, heat flux, electrical resistivity, and hydrostatic water pressure. There are also more than 630 sampling points, allowing physicochemical analyses of water and gases within the soil. Outside the hillslopes, water storage in the soil is accounted for through 10 large load cells for each hillslope whereas discharge is monitored by a combination of tipping buckets and electromagnetic flowmeters. Above ground, there are more than 50 sensors in each bay to monitor the enclosed atmosphere by measuring temperature, relative humidity, wind speed and direction, and radiation fluxes at different heights (Fig. 1b). Precipitation is not measured directly but precisely controlled by the irrigation system. The most recent data acquired in LEO can be visualized in http://biosphere2.org/research/leo-data.

**Fig. 2:**
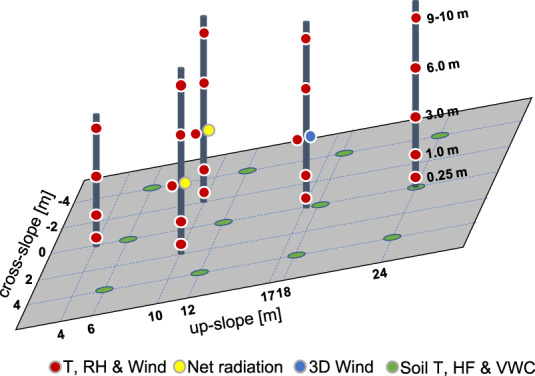
Instrument locations on each LEO artificial hillslope. Soil sensors are at 5 cm depth. T is temperature, RH is relative humidity, HF is soil heat flux, and VWC is Volumetric Water Content. Net radiation includes the four radiation fluxes.

Although all the monitored variables in LEO are valuable for the scientific community, the scope here is to provide data that helps to improve our understanding of the soil-atmosphere interactions. Careful processing, quality control, and then sharing of other data, including that from more than 3,000 sensors buried deeper in the soil, are left to future efforts. Hence, this dataset^30^ compiles part of the LEO measurements in the three individual hillslopes of (1) meteorological variables above the hillslopes’ surface, (2) soil moisture, heat flux and temperature of the soil near the surface, (3) precipitation, discharge and water storage aggregated for the entire hillslope, and (4) meteorological variables outside LEO from an automatic weather station. A basic description of each instrument used for the measurements included in this dataset is available in Tables 1, 2, and 3, while their locations within each bay are shown in Fig. 2.

**Table 1:** Summary of the above ground instrument’s specifications.

Variable (code)	Instrument	Qty per bay	Range of measurement	Uncertainty	Resolution	Additional information
Wind speed (WS)	Davis Instruments *DVI7911*	24	0.5 ms^−1^ to 89 ms^−1^	1 ms^−1^ or 5%	0.1 ms^−1^	Solid state magnetic sensor.
U-wind (U)	Campbell Scientific *CSAT3*	1	−60 ms^−1^ to 60 ms^−1^	Offset 0.08 ms^−1^ Gain 2%/3%/6% for wind within 5°/10°/20° of horizontal	0.001 ms^−1^	3D sonic anemometer. Set at sample rate of 60 Hz.
V-wind (V)						
W-wind (W)			−8 ms^−1^ to 8 ms^−1^	Offset 0.04 ms^−1^	0.0005 ms^−1^	
Air temperature (T)	Vaisala *HMP60*	24	−40 °C to 60 °C	• 0.5 °C when 10 °C < T < 30 °C • 0.6 °C when −40 °C < T < 10 °C or 30 °C < T < 60 °C	0.1 °C	
Relative humidity (RH)			0% to 100%	If 0 °C < T < 40 °C • 3% (RH) when 0% < RH < 90% • 5% (RH) when RH > 90% If −40 °C < T < 0 °C • 5% (RH) when 0% < RH < 90% • 7% (RH) when RH > 90%	0.1% (RH)	
Downward longwave radiation (DLW)	KippZonen *CNR4*	2	−250 Wm^−2^ to 250 Wm^−2^ of net radiation between the sensor and the external source	• Non-linearity < 1% • Temperature dependence < 4% when −10 °C < T < 40 °C • Tilt error < 1% • Offset < 6 Wm^−2^ • Non-stability < 1% • Spectral selectivity < 5% Total uncertainty < 10% of the daily total	<0.1 Wm^−2^	Wavelengths between 4.5 and 42 𝞵m, field of view 180° (150°) for downward (upward). Offset due to LW emitted by the sensor is accounted for using an internal thermistor.
Upward longwave radiation (ULW)						
Downward shortwave radiation (DSW)			0 Wm^−2^ to 2000 Wm^−2^	• Non-linearity < 1% • Temperature dependence < 4% when −10 °C < T < 40 °C • Tilt error < 1% • Offset < 15 Wm^−2^ • Directional error < 20 Wm^−2^ • Non-stability < 1% • Spectral selectivity < 3% Total uncertainty < 5% of the daily total	<0.1 Wm^−2^	Wavelengths between 0.3 and 2.8 *μ*m. Field of view 180° (150°) for downward (upward).
Upward shortwave radiation (USW)

**Table 2:** Summary of the in-soil instrument’s specifications.

Variable (code)	Instrument	Qty per bay	Range of measurement	Uncertainty	Resolution	Additional information
Soil heat flux (SHF1, SHF1sc)	Campbell Scientific (Hukseflux) *HFP-1*	12	−2000 Wm^−2^ to 2000 Wm^−2^	• Temperature dependence <0.1%/°C • Thermal conductivity dependence 7%/(W/(mK)) • Non-stability <1%/yr • Factory calibration uncertainty <3%	<0.1 Wm^−2^	Thermopile. At 5 cm depth.
	Campbell Scientific (Hukseflux) *HFP-1SC*	12				
Soil temperature (ST)	Campbell Scientific *TCAV*	12	−40 °C to 50 °C	Not reported	0.1 °C	Thermocouple. At 5 cm depth.
Volumetric water content (VWC)	Decagon *5TM*	12	0% to 50%	<2% (VWC) after soil specific calibration.	0.08% (VWC)	Apparent dielectric permittivity (ADP). At 5 cm depth.

The atmospheric sensors are located on five retractable masts at 0.25, 1, 3, 6, and 9–10 m above ground, with the exception of the mast at 4 m from the bottom of the hillslope where only the four lowest levels are present. At each level along the masts (Fig. 2), air temperature, relative humidity, and wind speed and direction are measured. On the two masts located at 10 m from the bottom of the hillslope, there are 4-channel radiometers measuring downward and upward longwave and shortwave radiation fluxes. In addition, a 3D sonic anemometer is located on the mast at 17 m from the bottom of the hillslope. Specifications for the above ground instruments are listed in Table 1. All masts are lifted during each rain event to avoid interference with the rain and nonuniform erosion of the soil through dripping. When the masts are lifted, most of the above ground measurements are not usable, as the location and orientation of the sensors are changed. Only the topmost measurements of temperature and relative humidity show some temporal consistency during such rain periods as they do not have much dependence on the orientation of sensors and their location is only slightly modified.

Among the sensors buried in the soil, there are measurements of near surface (at 5 cm depth) soil heat flux, soil temperature, and soil volumetric water content which are also included in the dataset (Fig. 2, Table 2). Heat flux is measured with two independent instruments at each location, HFP-1 and HFP-1SC.

Precipitation, discharge and water storage are also monitored in LEO, but they required additional processing, which was performed and explained in the following paragraphs. Details of the instruments and the aforementioned hillslope-scale quantities are available in Table 3.

**Table 3:** Summary of hillslopes instrument’s specifications.

Variable (code)	Instrument	Qty per bay	Range of measurement	Uncertainty	Resolution	Additional information
Precipitation (RRate)	Seametrics *EX81*	1	0.5 mmh^−1^ to 160 mmh^−1^	1% of full scale	—	Electromagnetic flowmeter, measure the irrigation flux not actual precipitation. Stored as rain rate.
Mass load (Mass)	Honeywell *Model 3130*	10	0 kg to 150,000+ kg	• Non-linearity 0.2% • Hysteresis 0.2% • Repeatability 0.05% • Temperature dependence offset 0.005%/°C • Temperature dependence gain 0.005%/°C • Zero balance 1%	—	
Relative water storage (RelS)	Derived	1	0 mm to 300 mm	—	—	See text for description of the computation.
Low flow discharge (QLow)	NovaLynx *260-2501-A*	6 - 7	0 mmh^−1^ to 0.6 mmh^−1^	2% at 0.012 mmh^−1^	<0.0001 mmh^−1^	Empirically calibrated, underestimates high flows.
High flow discharge (QHigh)	Seametrics *PE102*	6 - 7	0.025 mmh^−1^ to 2.5 mmh^−1^	1% or 0.003 mmh^−1^	<0.001 mmh^−1^	Very low accuracy for flows lower than 0.025 mmh^−1^.
Total discharge (QRate)	Derived	1	0 mmh^−1^ to 15 mmh^−1^	—	<0.001 mmh^−1^	See text for description of the computation.

Precipitation is not directly measured but highly controlled through a complex irrigation system. The total volume of water flowing through the irrigation main line is recorded and then converted to rain rates by dividing the water volume by the area of the hillslopes (330 m^2^) and by the time aggregation period in hours, to be included in this dataset. Droplet size distributions, terminal velocities of the droplets, and spatial homogeneity of the precipitation have been studied in the past^15^, showing that the irrigation system is able to produce rain droplets that achieve velocities close to that of natural rain. The spatial distribution has coefficients of variation between 0.2 and 0.7 with more homogeneous distributions occurring at higher rain rates.

LEO’s discharge is routed through a porous plate at the seepage face (11 m^2^) to six dividers and flow through a SeaMetrics PE102 flow meter (for high flow discharge) and then through a NovaLynx 26-2501-A tipping bucket gauge (for low flow discharge), both of which are included in this dataset. The Center hillslope had developed a leak at the bottom of the hillslope and additional flow meter and tipping bucket gauge were added to account for this in the total discharge. Flow meters have a low accuracy for flows below the equivalent to 0.025 mmh^−1^, while tipping buckets tend to underestimate the high flows. Our dataset also includes the computed total discharge as a reference. This quantity was calculated as the sum of the flows of the best available measurement, in each divide, based on the discharge rate. The flow meter (tipping bucket) value was selected for flows higher (lower) than 0.025 mmh^−1^ if both measures passed the quality control or whichever is available if only one passed the quality control. For this computation, a zero value was assigned to a divider when there was no discharge flow or measurement were missing or below the range.

Our dataset also includes measurements from 10 Honeywell Model 3130 load cells on each hillslope to monitor the total water storage. Relative water storage was computed by adding the weights from the 10 load cells (only when all of the measures passed the quality control) and then subtract the total mass weight from the lowest water storage content during the time period of this dataset, occurred on 6 November 2016. Assuming a water density of 1,000 kgm^−3^, the results were divided by the slope area (330 m^2^) to obtain the relative water storage content in mm which is also included in the dataset.

Although the greenhouses in LEO allow the control of many variables, some of them are still impacted by the external conditions. For instance, solar radiation inside the bays is directly dependent on the amount of solar radiation outside LEO and indoor temperature is highly impacted by the outside temperature. Hence, data from a weather station WeatherHawk 710 located outside the building were also included. Pressure, solar radiation (300–1100 nm), temperature and relative humidity of the air, are the prevalent variables impacting the internal environment of LEO but precipitation, wind speed and wind direction were also included for completeness.

### Experiments and precipitation control in LEO

Several specific experiments have been conducted in LEO which has led to particular rain patterns. In the provided dataset, there were two extensive tracer experiments carried out by the end of 2016 and in mid-2019.

The first extensive experiment was a 28-day tracer experiment conducted from 1 to 28 December 2016. The experiment was designed to observe the transit time distributions (TTDs) and the StorAge Selection (SAS) functions^31^, which are system-scale hydrologic transport signatures. Those functions were directly observed using the experimental protocol PERiodic Tracer Hierarchy (PERTH) method^32^. The method required driving the hillslopes to a periodic steady state. Therefore, the hillslopes were irrigated with two 3-hour pulses of 12 mmh^−1^ at 7-hour intervals every 3.5 days.

Before this experiment, no irrigation was performed within the period covered by this dataset until 6 November 2016, leading to the driest period recorded in those hillslopes. After this experiment was concluded, no irrigation was performed for about 4 months. In order to support the biogeochemical dynamic of the soil, a quasi-regular irrigation sequence, with precipitation almost every two weeks but with a few longer dry periods in between was performed until late June 2019 when the next major experiment started.

The second major experiment was conducted from 24 June to 16 August 2019, with only 7 days within this dataset. Its goal was to test a new TTD estimation method, which is not limited to a periodic steady state. The irrigation sequence was generated stochastically using a rainfall generator^33^. During this experiment, the total irrigation amount was about 750 mm with a mean irrigation rate of about 5.3 mmh^−1^. The mean duration of the irrigation pulses was 2.5 hours, and the mean inter-irrigation time was about 17 hours. Due to the complicated irrigation sequence, the mast operation was different from its regular operation and the corresponding times when masts were lifted were flagged in the quality control companion files.

### Instruments calibration and uncertainty

All the instruments were calibrated in the factory by the manufacturers, some of which have unique calibration coefficients that are applied before storing the data. The uncertainty reported in Tables 1, 2, and 3 correspond to that reported by the manufacturers. Additional uncertainties arise from different sources such as the data-acquisition devices, numerical roundoff, and dependence on other environmental conditions, among others.

The DVI7911 anemometers are known to have a very low accuracy for wind speeds below 0.5 ms^−1^ which occurs within LEO more than 99% of the time. Their corresponding wind vanes have even lower accuracy for such wind speeds and hence they were not included in this dataset. The CNR4 net radiation sensor is composed of 2 pyranometers and 2 pyrgeometers, each of them having unique calibration coefficients that are applied before storing the data. They also have internal temperature sensors that automatically compensates for thermal drifts.

For the volumetric water content, a calibration curve was developed specifically for the LEO soil through lab experiments based on four 5TM sensors buried in a sample of the same basaltic material with the same bulk porosity. This fitting curve allows to derive the volumetric water content from the directly measured dielectric permittivity, with a 95% confidence. The custom calibration was applied by the factory for Biosphere 2.

The EX81 flowmeter sensors use a calibration coefficient dependent on the material and size of the irrigation piping and provides the total volume of water sprayed by the irrigation system. This does not account for the small losses of water that fall outside the hillslopes’ surface nor the evaporation from the rain drops that occurs before they reach the surface. Hence, the precipitation is slightly overestimated by this measure. To reduce the uncertainty in the total precipitation rates, the changes in water storage content derived from the load cells and mass conservation can also be used.

The PE102 flowmeters have a low flow cutoff of 0.025 mmh^−1^, hence the flow is then routed to the tipping buckets which in contrast are known to underestimate the high flows. The Novalynx conversion rate between tip pulses and volume of water was derived empirically through manual calibration. The total discharge computed for this dataset has a larger uncertainty due to the assumption of no flow when data were missing or below the range. Therefore, a more detailed filling of the missing data is recommended for case studies.

### Data aggregation

The data were typically logged every 15 minutes but during specific experiments where high temporal resolution was required, the data were logged every minute for many sensors. Hence, in order to homogenize the series, all the data were aggregated/averaged to 15-minute intervals and labeled with the time for the end of the aggregation period.

## Data Records

This dataset^30^ is available in comma separated files. For the data inside LEO, there is one file for each variable and bay with the name BAY_VARIABLE.csv, where BAY is one of the three bays, i.e. East, Center or West and VARIABLE is the variable code listed in Tables 1, 2, and 3. The first column of each file provides the time corresponding to the end of the aggregation period for each record in the format YYYY-MM-DD HH:MM:SS (Mountain Standard Time) and the subsequent columns provide the time-aggregated value for the variable as measured by the sensor identified in the header row using the unit listed in the range of measurement in Tables 1, 2, and 3. The sensor naming convention, BAY_VARIABLE_UP_CROSS_LVL, is comprised of the bay identifier, the variable code, and three numerical identifiers of position, where BAY and VARIABLE are as defined before, UP and CROSS are the coordinates in meters for the up-slope and cross-slope directions respectively, measured from the center of the bottom edge of each hillslope, as illustrated in Fig. 2. LVL is an integer with a value of 0 for the instruments with a single available level (i.e., ST, VWC, SHF, DSW, USW, DLW, ULW, U, V, W, RRate, QHigh, QLow, QRate, Mass, and RelS) and from 1 to 5 for the sensor heights in the masts for multilevel data (lowest to highest).

For each variable and bay there is also a file containing a quality control (QC) flag code, as explained in the Technical Validation section below. Each file is named BAY_VARIABLE_qc.csv and has the same dimensions, records and format as the data record files but instead of the aggregated data, an integer QC flag code is provided (Table 4).

**Table 4:** Quality control codes. Specific details of the quality control process are in the text.

Status	Code	Bit	Description
Good	0	—	Data has passed all the quality controls
Missing	1	1	There is no record available for the 15-minute aggregation period.
Below range	2	2	Data value is below the expected range
Above range	4	3	Data value is above the expected range
Outlier	8	4	Data value deviate excessively from the mean of the values from the same sensor during a 30 days window.
Temporarily Inconsistent	16	5	Difference between consecutive records of a given sensor highly deviates from the mean of such differences during a 30 days window.
Spatially Inconsistent	32	6	Data value is responsible for an anomalous high spatial standard deviation in that aggregation time, as compared to the mean spatial standard deviation of the records within a window of 30 days.
Masts lifted	64	7	Any data within the time window covering from 60 minutes before the irrigation period to 60 minutes after.
Manually flagged	128	8	Data flagged after manual inspection. Mostly due to periods of anomalous nearly constant values.

Finally, the files EXTAWS.csv and EXTAWS_qc.csv provide the data and the QC flag codes for the automatic weather station located outside LEO respectively. The format is similar to the aforementioned files with the first column corresponding to the time of the end of the aggregation period for each record and the subsequent columns corresponding to the time-aggregated values for each variable or the QC flag code respectively. The header row indicates the variable in each column; the unit of measurement for each variable is the same as in the previous files and for pressure (P) is hPa. The QC flag codes are the same as defined in Table 4. Although the EXTAWS files span the same time period as the LEO data, the first non-missing record correspond to 18 February 2016 12:30 (Mountain Standard Time; or 19:30 UTC).

All the files contain exactly one row for each aggregation period of 15 minutes (140,257 records per sensor), even if no observations are available for such time. The missing values were filled with the value −9999.9. The quality control files have no missing values.

## Technical Validation

### Quality control overview

Although the quality and calibration of the sensors were carefully considered and protocols to avoid problems in the measurements have been followed, data were still prone to errors. Hence, an automated data quality control has been performed to account for missing values, values out of the range, outliers, temporal inconsistency and spatial inconsistency. No data were deleted from the dataset but a companion file with QC flag codes was included. Additionally, during the irrigation periods (rain), the masts were lifted and hence the above ground sensors were moved from their original locations and consequently flagged. Finally, data for some sensors were manually flagged to account for obvious problems.

The different quality control codes are compiled in Table 4. They were chosen to be a power of 2, to mark one specific bit in its binary representation. As errors in the data could be attributed to more than one type, a QC code was assigned to each record as the sum of all the codes that apply to the specific errors, or equivalently the error code has marked each bit corresponding to the sources of error in its binary representation. A value of 0 means that this record has passed all the quality controls. Different sources of error were analyzed sequentially, excluding the data flagged in the previous steps.

### Missing and constant data

Missing data records were flagged with a value of 1 (or equivalently the first bit of the QC flag was marked). During some periods of time, the values stored for some sensors were found to be frozen or remained constant. For this reason, a search in the data looking for at least 4 hours of constant values for the air temperature, relative humidity, volumetric water content, soil temperature, and soil heat flux was performed to flag the corresponding records as manually flagged by adding 128 to the QC code or equivalently the eighth bit being marked; while for radiation variables, constant data was searched for periods of 24 hours. Wind, precipitation, discharge flows, mass and relative water storage variables were excluded from this check because time periods with constant or nearly constant values are possible for these variables in LEO.

### Values out of range

Each record was quality controlled to ensure that the value is within the plausible range of measurements for that sensor within the LEO bays; minimum (R_min_) and maximum (R_max_) of the corresponding range for each variable/instrument are listed in Table 5. Whenever the data value was lower than R_min_, the QC code was incremented by 2 (or equivalently the second bit was marked). If the data value was higher than R_max_, the QC code was incremented by 4 (or equivalently the third bit was marked). It is important to note that it is not uncommon for temperature records on the topmost sensors to reach about 70 °C. Although the specifications of the sensors indicate that the valid range of measurements is up to 60 °C, there is no evidence of those values to be wrong, and hence they can still be used with caution.

**Table 5:** Quality control coefficients.

Variable (code)	R_min_	R_max_	k_o_	k_t_	k_s_	k_m_
Wind speed (WS)	0.5 ms^−1^	15 ms^−1^	10.0	—	0.1	7.0
U-wind (U)	−10 ms^−1^	10 ms^−1^	10.0	10.0	—	—
V-wind (V)	−10 ms^−1^	10 ms^−1^	10.0	10.0	—	—
W-wind (W)	−8 ms^−1^	8 ms^−1^	10.0	10.0	—	—
Air temperature (T)	−10 °C	70 °C	4.0	7.0	0.1	6.0
Relative humidity (RH)	0%	100%	4.0	7.0	0.1	7.0
Downward longwave radiation (DLW)	0 Wm^−2^	1200 Wm^−2^	10.0	7.0	—	—
Upward longwave radiation (ULW)	0 Wm^−2^	1200 Wm^−2^	10.0	7.0	—	—
Downward shortwave radiation (DSW)	0 Wm^−2^	1200 Wm^−2^	10.0	—	—	—
Upward shortwave radiation (USW)	0 Wm^−2^	300 Wm^−2^	10.0	—	—	—
Soil heat flux (SHF1, SHF1sc)	−2000 Wm^−2^	2000 Wm^−2^	4.0	7.0	0.1	5.0
Soil temperature (ST)	0 °C	80 °C	3.0	6.0	0.25	5.0
Volumetric water content (VWC)	0%	50%	6.0	—	0.1	5.0
Precipitation (RRate)	0 mmh^−1^	80 mmh^−1^	—	—	—	—
Mass load (Mass)	30000 kg	95000 kg	10.0	—	—	—
Relative water storage (RelS)	0 mm	300 mm	10.0	—	—	—
Low flow discharge (QLow)	0 mmh^−1^	0.4 mmh^−1^	—	—	—	—
High flow discharge (QHigh)	0 mmh^−1^	0.4 mmh^−1^	—	—	—	—
Total discharge (QRate)	0 mmh^−1^	2 mmh^−1^	—	—	—	—

### Lifting of the masts

The masts holding the above ground instruments were lifted a few minutes before each controlled rain event and were kept in a horizontal position up to a few minutes after the rain has ended. Hence, records for each above ground sensor during a time window extending 60 minutes before to 60 minutes after any rain period, were flagged with the code corresponding to “Masts lifted” by adding 64 to its corresponding QC code or equivalently turning on the seventh bit in its binary representation. During the last 7 days of the period covered by this dataset, a tracer experiment with randomly generated sequences of rain was performed. This led in periods with the masts lifted beyond the usual window, consequently the corresponding records were manually flagged as “Masts lifted”.

### Outliers

Individual records that highly deviated from the mean usually, but not always, correspond to errors in the measurements. Reasons for such values require a case by case analysis beyond the scope of this article. A variable/sensor-type specific threshold was defined here (*k*_*o*_ in Table 5), based on empirical trials to define an outlier. Note that even after defining such a threshold, it is still not possible to indicate that the outliers are all incorrect data. Hence, a case by case outlier analysis is advised if the use of the data could be affected by extreme values. The QC procedure for this outlier detection analysis is as follows: for each sensor, the centered running mean (*μ*_*r*_) and centered running standard deviation (${\sigma }_{r}$) with a time window of 30 days for the entire time series were computed. Then, for any value outside the range ${\mu }_{r}\pm {k}_{o}\cdot {\sigma }_{r}$, the corresponding QC code was incremented by 8 (equivalently, the fourth bit was marked). As the occurrence of precipitation and discharge is sparse, an analysis in this way is not possible, and hence this test was not applied to these variables.

### Temporal consistency

In an enclosed environment, sudden changes of atmospheric or soil parameters are not expected for most variables. Hence, when the change in the value between consecutive aggregation periods is high, it is reasonable to think that some error in the measurement is possible. However, it is not easy to define how large must be for such change to be considered anomalous. Therefore, after several trials, a specific threshold was defined for each variable/sensor-type (*k*_*t*_ in Table 5). The procedure was as follows: for every sensor the differences between any record and the record for the previous aggregation period were computed (∇), then the centered running mean (${\mu }_{r}^{\nabla }$) and the centered running standard deviation (${\sigma }_{r}^{\nabla }$) for such differences were computed with a time window of 30 days, any record would be flagged as temporarily inconsistent when that difference is out of the range ${\mu }_{r}^{\nabla }\pm {k}_{t}\cdot {\sigma }_{r}^{\nabla }$. For the flagged records, the QC flag code was incremented by 16 (equivalently, the fifth bit was marked). This QC check was not applied to precipitation, wind speed, shortwave radiation, volumetric water content, mass, water storage content, nor discharge because such sudden changes for these variables are possible. For precipitation, water content, and discharge this is expected during irrigation periods. Shortwave radiation fluxes also have abrupt changes at sunrise and sunset or during cloud passages.

### Spatial consistency

It is expected that in an enclosed environment with sensors very close to each other, the measurement of any given variable at the same vertical level present a very high correlation and values for the same aggregation period are expected to be very similar. In order to identify the data that do not present such spatial consistency, a two-step procedure was applied:

1. The standard deviation between all the sensors for a given variable at the same vertical level was computed for each aggregation time (*s*), the centered running mean value of this new time series was also calculated with a 30 days window (${\mu }_{r}^{s}$). With those values, the aggregation times when *s* was higher than a variable-specific constant (*k*_*m*_ in Table 5) times the mean standard deviation ${\mu }_{r}^{s}$ were selected for further analysis. Those selected aggregation times show a “high” inter-location standard deviation, suggesting that the value for at least one of the locations is very different compared to others at the same level for that time; hence, the second step is devoted to identify which value contributes to such large standard deviation.
2. If the standard deviation recalculated after removal of one specific sensor (*s*_−1_) is highly reduced, we could conclude that the value for that sensor was very different to the others and hence it was flagged. The criterion used for such decision was to identify the sensor that after removal reduces the standard deviation to less than a variable/sensor-type specific fraction (*k*_*s*_ in Table 5) of the original standard deviation with all the sensors for that level.

In summary, any record for an aggregation time when $s > {k}_{m}\cdot {\mu }_{r}^{s}$ where ${s}_{-1}/s < {k}_{s}$ were flagged as spatially inconsistent adding 32 to the QC flag code (equivalently, the sixth bit was marked). This check was performed for wind speed, air and soil temperatures, relative humidity, volumetric water content and soil heat flux, as such variables have more than the three sensors at the same vertical level, as required by this method.

### Checks for the external weather station

The data from the external weather station were also quality controlled. Wind direction, was marked as manually flagged (128 added to QC code, or the eighth bit marked) every time the wind speed was lower than 0.5 ms^−1^, due to the low accuracy of the sensor below such threshold. For wind speed the high extreme of the range was set to 40 ms^−1^, instead of the 15 ms^−1^ used for the inside LEO instruments, as higher values are possible when measured in the exterior. Spatial consistency was not possible to be assessed with just one location, hence that check was not performed. Temporal consistency and outlier checks were performed only for relative humidity, temperature and atmospheric pressure with values for the outlier threshold k_o_ of 5.5, 3.5, and 4.0 respectively, while the temporal threshold k_t_ was set to 10.0 for all of them.

### Quality control results

After the application of the previously described QC checks total, missing, flagged and non-flagged number of records were accounted for and are shown in Table 6. Only about 0.2% of the wind speed records are non-flagged, this is mostly due to speeds below the threshold of 0.5 ms^−1^, which is expected for indoor measurements; therefore, wind direction is not reliable and it was not included in the dataset. For the case of the 3D components of the wind a much higher percentage of the records passed the QC as the sensor is much more reliable for low wind speeds; most of the flagged records correspond to missing values, mainly from the sensor located in the West Bay where frequent and long outages occurred. Beyond the missing values, the most common cause for flagged records in the 3D wind components was the lifting of the masts.

**Table 6:** Summary of the quality control results.

Variable (code)	Total # records	% Good records	Missing # records	Total # flagged records	Additional information
Wind speed (WS)	10,098,504	0.2%	1,171,092	10,083,163	Most of the non-missing data with values below 0.5 ms^−1^
U-wind (U)	420,771	67.1%	131,470	138,515	Most of the flagged data were missing from the West bay and when masts were lifted.
V-wind (V)	420,771	67.1%	131,470	138,293	Most of the flagged data were missing from the West bay and when masts were lifted.
W-wind (W)	420,771	67.1%	131,470	138,645	Most of the flagged data were missing from the West bay and when masts were lifted.
Air temperature (T)	10,098,504	85.4%	950,082	1,475,664	Center and West had a period of about 6 months during 2016 with missing data.
Relative humidity (RH)	10,098,504	85.4%	950,082	1,477,950	Center and West had a period of about 6 months during 2016 with missing data.
Downward LW radiation (DLW)	841,542	83.1%	80,876	142,117	About 45,000 records showed unrealistic constant values for more than 24 hours.
Upward LW radiation (ULW)	841,542	82.9%	80,875	144,005	About 45,000 records showed unrealistic constant values for more than 24 hours.
Downward SW radiation (DSW)	841,542	40.7%	116,204	499,464	About 350,000 records with slightly negative values, most could be assumed to be zero.
Upward SW radiation (USW)	841,542	70.9%	80,876	244,887	About 125,000 records with slightly negative values, most could be assumed to be zero.
Soil heat flux (SHF1)	5,049,252	86.8%	398,449	668,882	In Center Slope, sensor in position 0_12_0 was flagged for the entire period.
Soil heat flux (SHF1sc)	5,049,252	86.8%	398,447	668,629	In Center Slope, sensor in position 0_12_0 was flagged for the entire period.
Soil temperature (ST)	5,049,252	89.2%	413,297	547,196	Variability in the mid-row of sensors within the East Bay was significantly lower than in the sides.
Volumetric water content (VWC)	5,049,252	54.4%	1,880,722	2,304,654	Most of very dry periods were shown as missing data or with negative values instead of 0% VWC.
Precipitation (RRate)	420,771	87.8%	51,256	51,257	Missing data could usually be considered as 0 mmh^−1^
Mass load (Mass)	2,664,883	87.9%	234,600	323,511	Most of the non-missing records were negative values that could be assumed to be 0 mmh^−1^.
Relative water storage (RelS)	2,664,883	86.2%	296,347	368,825	Most of the non-missing records were negative values that could be assumed to be 0 mmh^−1^.
Low flow discharge (QLow)	420,771	89.6%	43,690	43,690	QC did not find any other anomalous value to flag after individual QLow and Qhigh were cleaned.
High flow discharge (QHigh)	4,207,710	91.0%	348,346	377,855	About 30,000 records were found to be out of the expected range.
Total discharge (QRate)	420,771	87.9%	50,509	50,793	Missing values arisen when at least one record was flagged on Mass variable.

Temperature and relative humidity records passed the QC checks in more than 85% of the cases. The missing records were mostly from an outage period of about six months during 2016 that affected those sensors in the Center and West bays and also from several shorter periods of constant values that were marked as manually flagged due to unrealistic constant values for at least 4 hours.

Longwave radiation has less than 12% of the data flagged with just a few of them for reasons other than missing values or the lifting of the masts. A more detailed analysis is advised for the longwave radiation data between late 2017 and mid 2018 in the West bay, as those data show a much larger dispersion than in any other period and bay. For downward shortwave radiation, only about 40% of the data were not flagged, with about 350,000 records showing slightly negative values (about other 40%) but, in most cases, they could be considered as 0 Wm^−2^ as they mostly occurred at night. In the case of upward shortwave radiation a similar situation occurs, but a 70% of the data passed the QC.

For the soil variables, both heat flux sensors (HFP1 and HFP1sc) at location 0_12_0 of the Center bay showed mostly anomalous values and hence all the records for such sensors were manually flagged. Soil temperature exhibits more than 89% of records passing the QC, with most of the flagged values being just missing records. More than 45% of the volumetric water content were flagged due to missing values or slightly negative values during the driest periods (most of the negative values could be interpreted as a value near 0% of VWC).

For precipitation, only one value was found to be above the range of expected measurements; although 12.2% of the precipitation data were missing, most of them could be assumed to be 0 mmh^−1^, as they mostly occurred during no-irrigation periods. About 13% of the data from the tipping buckets and flowmeters were QC flagged, mostly because of data being missing or negative, with only 308 and 16 records flagged due to other reasons. It is also reasonable to assume a flow of 0 mmh^−1^ for missing or below range values. The total discharge data would pass the QC checks when low or high discharge was available. In the case of mass measured from the load cells, there were about 350,000 missing data, while about 30,000 records were out of range. Flagged mass data resulted in about 50,000 missing records for relative water storage content while less than 300 records were flagged as outliers.

After the QC, several key variables of the energy and water cycles were aggregated at a daily time scale and spatially averaged, if applicable, for each of the three artificial hillslopes in LEO. Only the non-flagged data were used to prepare the plot, with the only exception of the radiation components set to 0 Wm^−2^ when they were flagged as below range. The spatial average data were computed using all the non-flagged 15-minute records for the applicable variable. These data were then used to compute the daily aggregated value when at least 75% of the daily data were available.

**Fig. 3:**
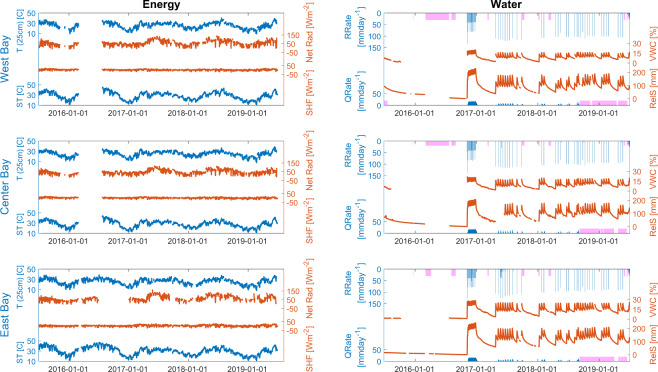
Time series of key variables in the three bays of LEO. Daily and spatially aggregated time series of soil temperature (ST; at 5 cm depth), air temperature (T; at 25 cm height), soil heat flux (SHF; at 5 cm depth), and net radiation (Net Rad; at 3 m height) are shown in the left panels, while discharge rate (QRate), rain rate (RRate), relative water storage content (RelS), and volumetric water content (VWC; at 5 cm depth) in the right panels.

The resulting aggregated time series in Fig. 3 show consistent day-to-day, seasonal, and interannual variabilities related to the energy cycle in the three hillslopes. The variability of water cycle variables is also consistent with that of the controlled precipitation. Figure 3 also shows an outage of approximately 6 months in the West and Center bays, during the driest period when no irrigation was performed.

Overall the dataset exhibits good quality. Indeed, a very low number of records were flagged due to reasons other than missing values, making it a suitable resource for case studies at high temporal resolution and very high spatial resolution.

## Usage Notes

This dataset does not represent a natural environment as most of the variables are heavily influenced by the greenhouses microclimate.

### Gap filling of the flagged data

In order to make use of the data, filling of the gaps due to QC flagged data could be required. The high density of the sensors for some variables, the redundant information for others, and their own behavior can be exploited to fill the gaps more accurately than in other environments.

For temperature and relative humidity of the air, and heat flux, temperature and volumetric water content of the soil, the measurements on any of the other sensors at the same level could provide a good estimate of the tendencies when data from just a few sensors are not available. If it is a short period of time, standard interpolation methods are still suitable.

Shortwave radiation must be 0 Wm^−2^ during night, when most of the records flagged as below range happened to occur. Tendencies of downward shortwave radiation among the two sensors in each bay are expected to be similar, but that assumption could not be valid for its upward counterpart as they are located over portions of the hillslopes with opposite slopes as could be seen in Fig. 1. For longwave radiation, the data flagged as below range also could be assumed as 0 Wm^−2^ in most of the cases, and it could be checked using the temperature measurements and the basic radiation laws.

Precipitation data could be adjusted, and its gaps filled, using mass conservation and the water storage content; although almost every missing precipitation record occurred during periods of no irrigation (except for the missing data during January 2018). Similar approaches could be also used for the discharge data.

### Quality control usage

Most of the flagged values do not necessarily represent bad data; instead they are not typical and require more detailed analysis. For data access and analysis, the users can use bitwise logical operators to identify specific sources of flagging. For instance, to identify if a record is flagged due to temporal inconsistency, bitwiseand(flagCode,16) = = 16 where 16 is the code for temporal inconsistent data and bitwiseand is the function to perform bit by bit AND operation that correspond to the programming language of the user.

## Data Availability

The processing of all the data was performed using R v3.3.2 within R Studio v1.1.447 for Mac. The library openair^34^ v2.0.0 was used for time aggregation, and the library caTools v1.17.1.1 was used to compute rolling means and rolling standard deviations of the time series. Figure 3 was produced with MATLAB^TM^ R2019b Update 4. The code is publicly available in figshare^35^. It contains all the functions to read, homogenize, and quality control the raw data files to produce the currently shared dataset. Also included are functions to read the data and QC flag codes on the final dataset in conjunction with the script used to produce the aggregated data for Fig. 3 that could be used as an example to process the data.
